# Exploratory Analysis on the Role of Video Tools and Multidisciplinary Healthcare Providers to Aid Counselling for Buprenorphine/Naloxone Induction Within the Evaluating Microdosing in the Emergency Department Study

**DOI:** 10.7759/cureus.83593

**Published:** 2025-05-06

**Authors:** Alyssa Kong, Viseth Long, Elle Wang, Anthony Lau, Jessica Moe

**Affiliations:** 1 Department of Emergency Medicine, St. Paul's Hospital, Vancouver, CAN; 2 Department of Emergency Medicine, Vancouver General Hospital, Vancouver, CAN; 3 Faculty of Medicine, University of British Columbia, Kelowna, CAN; 4 Department of Emergency Medicine, University of British Columbia, Vancouver, CAN; 5 Faculty of Pharmaceutical Sciences, University of British Columbia, Vancouver, CAN

**Keywords:** buprenorphine, medication for opioid use disorder, opioid agonist therapy, opioid use disorder, suboxone

## Abstract

Introduction

To address the rising deaths and hospitalizations related to opioid toxicity, buprenorphine/naloxone has been provided as a frontline treatment within the emergency department (ED), allowing increased access to opioid agonist therapies (OAT). However, several barriers exist toward initiating patients on buprenorphine/naloxone within the ED, such as shortages in healthcare staff and time limitations in the urgent ED environment. To overcome these barriers, we utilized counselling tools and engaged multidisciplinary healthcare providers to counsel patients for our study, Evaluating Microdosing in the Emergency Department (EMED). The primary objective of this analysis is to describe the proportion of healthcare providers counselling for buprenorphine/naloxone induction within the EMED study, before and after the implementation of a counselling video, and across different time intervals. Additionally, we aim to compare the completion status of enrollment when patients were counselled by different types of healthcare providers. By analyzing these factors, we aim to explore how collaboration with multidisciplinary healthcare providers and the use of video tools can impact buprenorphine/naloxone induction in the ED, thereby increasing accessibility of take-home OAT.

Methods

Data regarding the type of healthcare worker providing counselling for the EMED study was collected at Vancouver General Hospital from July 23, 2021, to December 31, 2023. The EMED study is an open-label, randomized controlled trial comparing microdosing and standard dosing take-home regimens. We analyzed 172 providers involved in counselling patients for the study. We stratified the data by month, week, and day of the week to assess trends in counselling frequencies.

Results

We found a statistically significant increase in the proportion of emergency physicians counselling after the implementation of the counselling video. Specifically, we found there was a statistically significant increase in the evenings. Furthermore, we found that pharmacists completed a greater proportion of counselling for buprenorphine/naloxone induction throughout the study period, and there was no statistically significant difference between the completion status of enrollment when counselled by either provider.

Conclusion

We can improve patient access to buprenorphine/naloxone induction counselling by utilizing an adjunct video tool and distributing workload between emergency physicians and clinical pharmacists. Future sites interested in providing take-home buprenorphine/naloxone kits may benefit from implementing video tools and involving clinical pharmacists to mitigate workflow burdens.

## Introduction

Since 2016, British Columbia has declared the opioid crisis a public health emergency, due to increasing deaths related to opioid overdoses [1]. To address this crisis, various efforts such as increasing opioid agonist therapy (OAT) accessibility in frequently visited healthcare settings, such as the emergency department (ED), have been evaluated [2,3]. Buprenorphine/naloxone (Suboxone) is an example of an OAT that has emerged as a front-line treatment for those with opioid dependencies, strongly encouraged by the guidelines set forth by the Canadian Medical Association [4,5]. This is due to the superior safety profile of buprenorphine/naloxone compared to other opioid agonist therapies, such as methadone, as buprenorphine/naloxone has a lower potential for causing respiratory depression and a reduced risk of overdose [6,7]. Studies demonstrate that providing buprenorphine in the ED significantly decreases illicit opioid usage, increases engagement with addiction treatment services, and is associated with reduced morbidity [8-10]. However, several barriers exist toward initiating patients on buprenorphine/naloxone in the ED, such as shortages in healthcare staff to support the initiation of buprenorphine/naloxone and time constraints for counselling patients regarding the safe use of buprenorphine/naloxone [5,11,12]. As a result, there is a need to optimize buprenorphine/naloxone induction in the ED [9].

To support the efforts of increasing OAT accessibility in the ED, healthcare providers, including pharmacists, have assumed expanded roles in addiction support [13]. At Vancouver General Hospital, clinical pharmacists facilitate buprenorphine/naloxone counselling through the Evaluating Microdosing in the Emergency Department (EMED) study, in addition to their regular consultations for appropriate therapies and dosage recommendations for patient care.

The EMED study is an ongoing randomized controlled trial comparing the effectiveness of buprenorphine/naloxone take-home induction regimens to examine: (1) the completion of the induction regimen and (2) the retention of patients on OAT at several sites across British Columbia and Alberta. To address the barriers associated with providing buprenorphine/naloxone in the ED, we involved the support of multidisciplinary healthcare providers and the use of counselling tools. The overarching objective of the present manuscript is to describe the impacts of these strategies on decreasing barriers toward buprenorphine/naloxone counselling within the EMED study. We report on the relative frequency of counselling provided by emergency physicians and pharmacists over the course of the EMED study at Vancouver General Hospital and compare the frequencies of emergency physician versus pharmacist counselling before and after the implementation of a counselling video. Additionally, we compare the frequency of counselling completed by emergency physicians and pharmacists by month of the year, day of the week, and hour of the day. Finally, we compare the completion rates of enrollment between different healthcare workers.

## Materials and methods

In the EMED study, we identified ED patients with opioid use disorder (OUD) who were eligible for buprenorphine/naloxone using screening criteria that assessed recent opioid use, absence of recent filled prescriptions of OAT (within five preceding days), and a positive score on the Rapid Opioid Dependence Score (RODS). Once determined eligible, we randomized participants into either microdosing or standard dosing induction, providing a five-day supply of buprenorphine/naloxone. During enrollments, clinical pharmacists and emergency physicians counseled participants for either induction method.

At Vancouver General Hospital, clinical pharmacists were available to counsel patients between 08:00 and 23:00 on weekdays and 13:00-21:00 on weekends, whereas the patients’ physicians or other physician colleagues experienced in addiction treatment were available to counsel throughout the entire 24-hour period. Emergency physicians were additionally provided with the option to use a video tool to supplement counselling when constrained for time, as counselling takes approximately 10-15 minutes.

The contents of the video included the following: medication dosing in each induction regimen, buprenorphine/naloxone risks, and follow-up clinic information. It was produced by the EMED study team with the input of the principal investigator, who is an emergency physician with additional addiction training, and a person with lived experience of opioid use. When the video was used to supplement clinician counselling, a trained study member showed the video to the patient. Subsequently, the patient’s emergency physician assessed the patient’s understanding of the information within the video, reaffirmed follow-up clinic information, and answered patient questions. To standardize counselling completed by the pharmacist or emergency physician, the study team also provided training to each provider group (led by the study principal investigator) and created a counselling checklist that was available to all providers, reinforcing important aspects of counselling. Providers also received a template for documenting information relayed to the patient. After counselling the patient (with or without the video), both provider groups were trained to ask if the patient had any questions to clarify the process. Study members were also present throughout the entire counselling procedure to ensure that all steps of counselling were completed for both provider groups. These study procedures ensured the content of patient counselling was standardized across both provider groups.

For this study, we conducted an exploratory analysis of counselling data from our EMED study. Therefore, we did not calculate an a priori sample size.

Data source and collection

We collected data regarding 172 counselling sessions for the EMED study at the Vancouver General Hospital site between July 23, 2021, and December 31, 2023. We analyzed the following variables: date of counselling, time of counselling, type of healthcare provider completing counselling, and completion status of enrollment. The usage of video counselling was not specifically recorded during the process. Data cleaning involved removing missing values and addressing any inconsistencies in data recording. We further organized data using Python in Jupyter Labs through Visual Studio Code to create data tables, alongside Tableau and Excel (Microsoft® Corp., Redmond, WA) to generate graphs. We determined the number of complete and incomplete enrollments for each type of healthcare provider by evaluating our study data stored on a Research Electronic Data Capture (REDCap) database hosted at the University of British Columbia [14,15].

Statistical analysis

To evaluate statistical significance, we used chi-square tests to compare the proportion of healthcare providers counselling for the study between notable date ranges and the completion status of enrollment, depending on the type of healthcare provider involved in counselling. To explore whether results varied by time of day, we conducted a sensitivity analysis stratifying the enrollments into morning (08:00-15:30) and evening (15:31-23:00) shifts. Additionally, we performed a logistic regression analysis to evaluate the effects that the counselling video, time of day, and day of the week had on counselling completion between the two types of healthcare providers. In the analyses, we used Nagelkerke’s R^2^ to determine how much of the variation in provider type is explained by the model.

## Results

**Figure 1:**
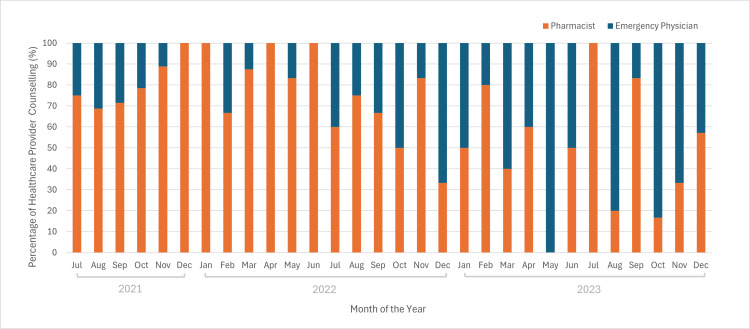
The proportion of pharmacists and emergency physicians providing counselling out of the total number of counselling sessions completed each month from July 2021 to December 2023.

Figure 1 demonstrates the proportion of pharmacists or emergency physicians providing counselling for buprenorphine/naloxone ED induction out of the total number of counselling events completed by healthcare providers each month from July 23, 2021, to December 31, 2023. We observed a downward trend in the percentage of pharmacists completing counselling over time, while emergency physicians exhibited an opposite, upwards trend. Notably, during the period spanning 2021-2022, pharmacists conducted a higher proportion of counselling compared to emergency physicians, with pharmacists providing counselling at a higher proportion of cases for 16 out of the 18 months. In comparison, pharmacists counselled more frequently than emergency physicians for only five months in 2023, and emergency physicians counselled more frequently than pharmacists for five months. For the remaining two months, emergency physicians and pharmacists provided counselling for an equivalent proportion of cases.

Table 1 demonstrates the number of cases in which pharmacists and emergency physicians provided counselling across different time periods. On May 05, 2023, we introduced a counselling video as an optional support tool for emergency physician counselling. We compared counselling rates between May 05, 2023, and December 31, 2023, to three prior periods: (1) eight months before the implementation of the counselling video, (2) 12 months before, and (3) the beginning of the study to May 05, 2023. The distribution of provider-led counselling video before and after video implementation was statistically significantly different, between July 23, 2021, to May 05, 2023, and May 05, 2023, to December 31, 2023 (chi-square=14.25, df=1; p<0.001). Similarly, compared to the 12-month period before the implementation of the counselling video, the distribution of pharmacist- and emergency physician-led counselling following its introduction remained significantly different (chi-square=5.29, df=1; p=0.02). However, no statistically significant difference was observed when comparing the distribution of counselling providers in the eight months before and after the implementation of the video (chi-square=0.85, df=1; p=0.36).

**Table 1:** The number of patients counselled by pharmacists or emergency physicians between specific date ranges, with a comparison between date ranges and reference period (May 05, 2023, to December 31, 2023) after introduction of the counselling video (chi-square). * p<0.05

Date Range of Counselling Competed	Pharmacist	Emergency Physician	p-value
May 05, 2023 to December 31, 2023	16	22	--
September 05, 2022, to May 05, 2023 (8 months prior)	14	12	0.36
May 05, 2022, to May 05, 2023 (12 months prior)	37	19	0.02*
July 23, 2021, to May 05, 2023 (from the beginning of study)	100	34	<0.001

Additionally, we conducted a sensitivity analysis to explore whether the distribution of provider types varied by time of day, using the same time periods in Table 2. We categorized shifts as morning (08:00-15:30) and evening (15:31-23:00), during which pharmacist and emergency physician hours overlap. Following the implementation, the proportion of patients counselled by physicians increased during evening shifts. This difference was significant across all pre-video comparison periods. However, we found no significant difference in morning shifts between pre- and post-video periods.

**Table 2:** Number of patients counselled by pharmacists or emergency physicians during morning and evening periods, before and after the introduction of the counselling video (chi-square). * p<0.05

Date Range of Counselling Competed	Time Period of the Day	Pharmacist	Emergency Physician	p-value
May 05, 2023, to December 31, 2023	Morning	10	3	--
	Evening	3	13	--
September 05, 2022, to May 05, 2023 (8 months prior)	Morning	4	2	0.64
	Evening	8	2	0.002*
May 05, 2022, to May 05, 2023 (12 months prior)	Morning	12	2	0.56
	Evening	23	7	<0.001*
July 23, 2021, to May 05, 2023 (from the beginning of study)	Morning	51	8	0.39
	Evening	47	13	<0.001*

As seen in Table 3, we used logistic regression to assess the effects of the counselling video implementation, time of day, and day of the week on the likelihood of emergency physician-led counselling. The model significantly predicted counselling likelihood (chi-square=25.07, p=0.002), with a Nagelkerke R² of 0.171, indicating that the model could account for approximately 17% of the variance in the outcome. Classification accuracy was 76.1%, with high specificity (93.6%) for identifying emergency physicians not providing counselling and lower sensitivity (32.1%) for emergency physicians who did during a counselling session. Video implementation was associated with increased emergency physician counselling (OR=3.31; 95% CI=1.53, 7.15; p=0.002) when controlling for time of day and day of the week.

**Table 3:** Logistic regression analyses of the association between video, time, and weekday on emergency physician and pharmacist counselling. * p<0.05

Variables	Coefficient	Standard Error	Odds Ratio	p-value
Emergency physicians				
Before and after video implementation	1.20	0.39	3.31	0.002*
Time	0.000	0.000	1.000	0.08
Sunday	--	--	--	0.13
Monday	-0.84	0.63	0.43	0.18
Tuesday	-1.55	0.63	0.21	0.01*
Wednesday	-1.76	0.74	0.17	0.02*
Thursday	-1.15	0.65	0.32	0.08
Friday	-1.30	0.70	0.27	0.06
Saturday	-0.52	0.64	0.60	0.42
Constant	0.59	0.68	1.81	0.38
Pharmacists				
Before and after video implementation	-1.07	0.38	0.34	0.005*
Time	0.000	0.000	1.000	0.06
Sunday	--	--	--	0.42
Monday	1.22	0.64	3.37	0.57
Tuesday	1.32	0.61	3.76	0.03*
Wednesday	1.22	0.66	3.38	0.06
Thursday	1.38	0.64	3.96	0.03*
Friday	1.36	0.67	3.88	0.04*
Saturday	1.04	0.65	2.82	0.11
Constant	-1.29	0.68	0.28	0.06

The logistic regression model for pharmacist counselling was also significant (chi-square=20.181, p=0.010), with a Nagelkerke R² of 0.131 and an overall accuracy of 63.5%. Sensitivity was high (81.9%) for identifying pharmacists who counselled, but specificity was lower (37.0%) for identifying those who did not. When controlling for time of day and day of the week, video implementation was associated with a decrease in pharmacist counselling (OR=0.34; 95% CI=0.16, 0.73; p<0.005).

**Figure 2:**
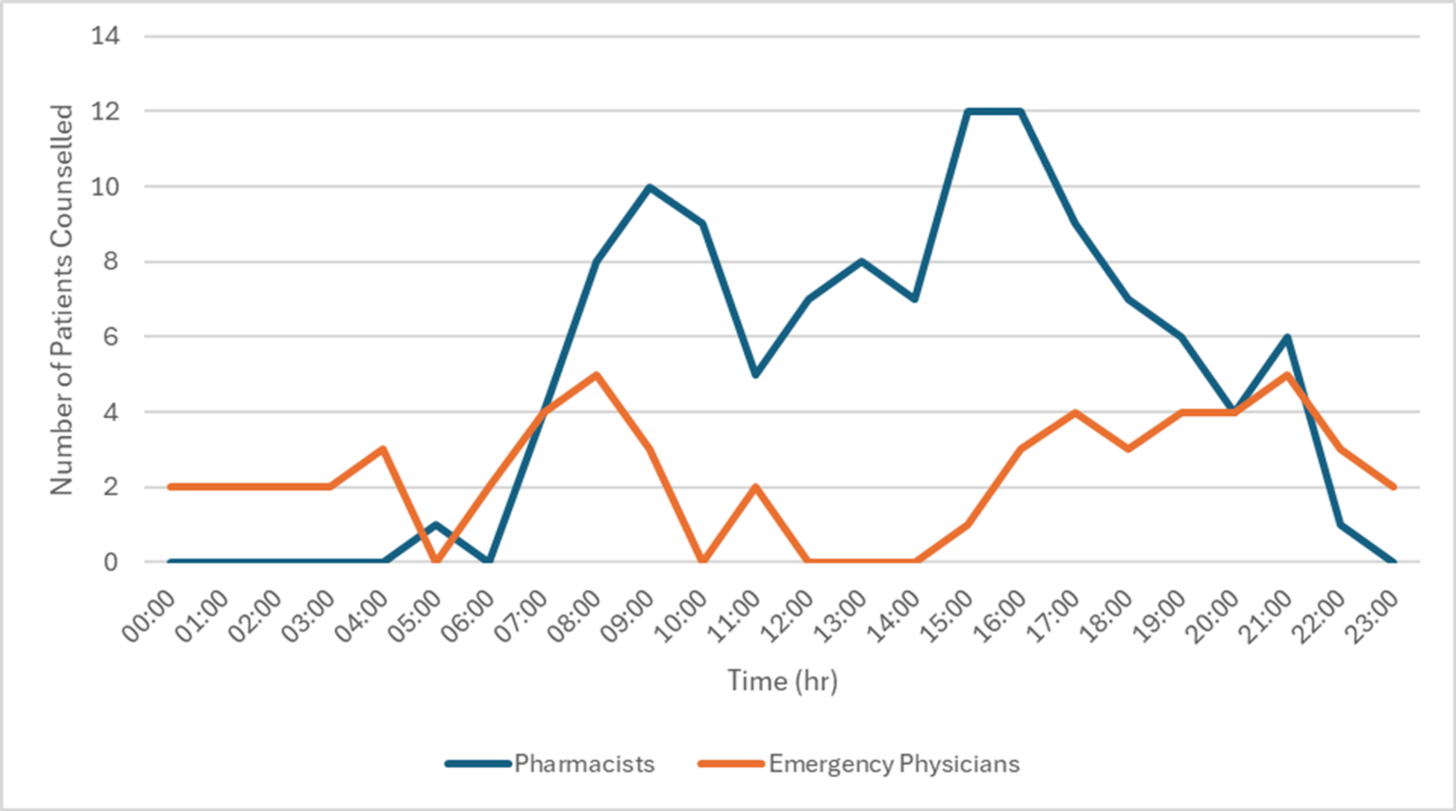
Total number of emergency physician and pharmacist counselling for the study by hour over a 24-hour period.

Figure 2 demonstrates the number of emergency physicians and pharmacists providing counselling by time of day, noted as hours within a 24-hour period. The times with the highest number of pharmacists completing counselling were 09:00 and 15:00-16:00. Conversely, the hours with the greatest number of emergency physicians completing counselling were 08:00 and 21:00, slightly earlier and a few hours later than the peak hours for pharmacists.

**Figure 3:**
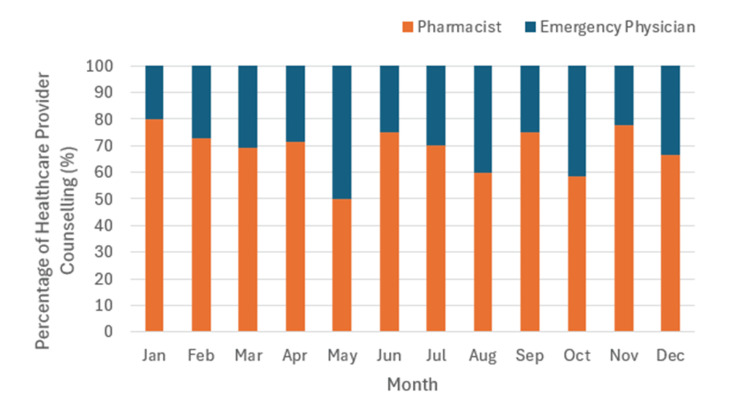
The percentage of patients who received counselling by emergency physicians and pharmacists by month of the year.

Figure 3 summarizes the monthly percentage of counselling completed by pharmacists and emergency physicians from July 23, 2021, to December 31, 2023. For all months except May, pharmacists counselled a greater proportion of patients than emergency physicians, with May showing equal contributions.

**Figure 4:**
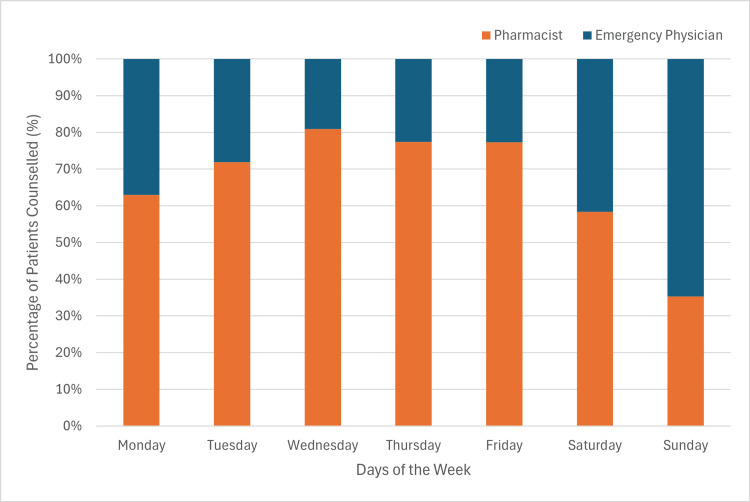
The proportion of patients counselled who received counselling by pharmacists and emergency physicians by day of the week (Monday to Sunday).

Figure 4 illustrates the percentage of pharmacists and emergency physicians counselling for the study by day of the week, from Monday to Sunday. From Monday to Saturday, pharmacists counselled a greater proportion of patients than emergency physicians, whereas on Sundays, emergency physicians counselled a greater proportion.

Table 4 explores whether the type of healthcare provider involved in counselling had subsequent impacts on the completion of enrollments. Between the two groups of providers, the difference in enrollment completion rates was not statistically significant (chi-square=2.49, df=1; p=0.11).

**Table 4:** The number of enrollments subsequently completed after the pharmacist or emergency physician provided counselling for the study and the proportion of incomplete enrollments out of total enrollments completed by the type of healthcare provider (chi-square). * p<0.05

Type of Healthcare Provider Involved in Counselling	Complete Enrollments	Incomplete Enrollments	Proportion of Incomplete Enrollments for Each Type of Healthcare Provider (%)	p-value
Pharmacist	111	5	4.3%	0.11
Emergency Physician	56	0	0%	

## Discussion

These findings highlight that the introduction of a counselling video may influence the frequency of buprenorphine/naloxone counselling in the ED. Figure 1 demonstrates an increase in emergency physician-led counselling and a decrease in pharmacist-led counselling over time. The counselling video may have contributed to this shift by enabling patients to review key information alongside a study team member, reducing the counselling workload for emergency physicians. In addition, when compared to the proportion of counselling completed by different types of healthcare providers over a 12-month period beforehand, the proportion of counselling completed by physicians and pharmacists following video implementation was statistically significantly different. However, when comparing the eight-month period before and after video introduction, we did not observe a significant difference, suggesting that the increased frequency of physician counselling was influenced by low physician counselling in the early months of the study. This pattern suggests that improved physician confidence and familiarity with buprenorphine/naloxone induction procedures may have influenced a trend toward greater physician engagement in counselling, which the counselling video further supported.

To further examine these trends, we plan to analyze emergency physician counselling patterns at St. Paul’s Hospital, a study site without pharmacist involvement. This will help isolate the counselling video’s impact on physician-led counselling. We also aim to assess whether provider type impacts counselling duration and overall enrollment time.

Our analysis assessing the impact of time and day of the week found that emergency physician-led counselling increased significantly during evening shifts following video implementation but not during mornings, likely due to pharmacist availability (Table 2). Clinical pharmacists are the primary counselling providers until around 20:00. After this period, the pharmacists are less available for patient care as they need to complete other end-of-shift tasks. This may explain the lack of statistically significant change in physician counselling during morning shifts following video implementation, as clinical pharmacists are readily available to provide counselling during the mornings. However, there was a statistically significant increase in emergency physician counselling in the evenings after the video was implemented. This suggests that the emergency physicians found the tool to be useful, allowing them to engage in more counselling sessions. As a result, care can be provided to patients more readily by either provider. Additionally, our logistic regression analysis, which controlled for time of day and day of the week, reinforced our finding that the video increased physician counselling.

Further analysis of counselling patterns (Figures 2-4) indicates that pharmacists consistently counselled a greater proportion of patients since study inception, particularly during their scheduled working hours (08:00-23:00 on weekdays; 13:00-21:00 on weekends). While emergency physicians increased their involvement over time, pharmacists remained key contributors, especially on weekdays. These trends underscore the integral role of pharmacists in ED-based buprenorphine/naloxone induction.

Table 4 demonstrates no statistical significance difference in enrollment completion based on whether pharmacists or emergency physicians conducted the counselling. This suggests that the type of healthcare provider involved in counselling does not impact the likelihood of successful enrollment. Despite differing responsibilities and availability within the ED, we found no significant difference between the rate of incomplete enrollments for patients counselled by either group. Therefore, we hypothesize that either healthcare provider can provide information in a manner that is acceptable to patients. Given the increased proportion of pharmacist-led counselling, these findings (Figures 2-4) reinforce the value of pharmacists in supporting buprenorphine/naloxone induction initiatives in the ED.

Limitations of the study

Our study has several limitations. Staffing constraints may have influenced counselling availability, as pharmacists have more restricted working hours compared to emergency physicians. Pharmacists are only available between 08:00-23:00 on weekdays and 13:00-21:00 on the weekends, making a direct comparison between pharmacist and emergency physician counselling rates more challenging. However, despite these shift differences, pharmacist schedules remained consistent throughout the study period, and emergency physician counselling increased significantly over time. Furthermore, our sensitivity analysis only examined counselling sessions completed during overlapping shift times for the two provider groups, allowing for a more direct comparison.

Another limitation is the absence of detailed records of actual video usage. Emergency physicians could counsel with or without the video, making it difficult to assess its direct impact. More specific data collection regarding the usage of the counselling video may allow for a stronger comparison by determining the frequency of counselling completed with or without the counselling video and exploring the impact of the video on actual counselling time. Additionally, our planned analysis of the usage of the counselling video at multiple hospital sites will assess whether the video is appropriate to supplement patient counselling compared to counselling completed entirely by a healthcare provider without video support. During future analyses, we will also explore the impact of patient age and disease severity on video usage and counselling outcomes.

Finally, our results are exploratory, and generalizability to other sites across Canada is limited by our analysis of data from a single site in Vancouver. Future research should explore the impact of video usage at multiple sites across various jurisdictions.

## Conclusions

In this article, we highlight the role of a video counselling tool and multidisciplinary healthcare providers in supporting buprenorphine/naloxone induction within the ED. Throughout the study, emergency physician involvement in counselling increased significantly, particularly in the evenings following the counselling video implementation, suggesting that the tool helped mitigate workflow constraints. Additionally, pharmacists played a key role in counselling, likely due to their greater availability to counsel, reinforcing their importance in buprenorphine/naloxone induction initiatives in the ED. Our results suggest a combined approach to counselling for buprenorphine/naloxone that offers a video adjunct with multidisciplinary counselling may facilitate greater access to standardized patient counselling when receiving a buprenorphine/naloxone induction in the ED.

## References

[1] Cheung A, Marchand J, Mark P (2023). Loss of life and labor productivity: the Canadian opioid crisis. Ann Am Acad Pol Soc Sci.

[2] Stone KD, Scott K, Holroyd BR (2023). Buprenorphine/naloxone initiation and referral as a quality improvement intervention for patients who live with opioid use disorder: quantitative evaluation of provincial spread to 107 rural and urban Alberta emergency departments. CJEM.

[3] Kelly TD, Hawk KF, Samuels EA, Strayer RJ, Hoppe JA (2022). Improving uptake of emergency department-initiated buprenorphine: barriers and solutions. West J Emerg Med.

[4] Koh JJ, Klaiman M, Miles I (2020). CAEP position statement: emergency department management of people with opioid use disorder. CJEM.

[5] Savage T, Ross M (2022). Barriers and attitudes reported by Canadian emergency physicians regarding the initiation of buprenorphine/naloxone in the emergency department for patients with opioid use disorder. CJEM.

[6] Bruneau J, Ahamad K, Goyer MÈ (2018). Management of opioid use disorders: a national clinical practice guideline. CMAJ.

[7] Bell JR, Butler B, Lawrance A, Batey R, Salmelainen P (2009). Comparing overdose mortality associated with methadone and buprenorphine treatment. Drug Alcohol Depend.

[8] D'Onofrio G, O'Connor PG, Pantalon MV (2015). Emergency department-initiated buprenorphine/naloxone treatment for opioid dependence: a randomized clinical trial. JAMA.

[9] Herring AA, Rosen AD, Samuels EA (2024). Emergency department access to buprenorphine for opioid use disorder. JAMA Netw Open.

[10] Wakeman SE, Larochelle MR, Ameli O (2020). Comparative effectiveness of different treatment pathways for opioid use disorder. JAMA Netw Open.

[11] Pourmand A, Beisenova K, Shukur N, Tebo C, Mortimer N, Mazer-Amirshahi M (2021). A practical review of buprenorphine utilization for the emergency physician in the era of decreased prescribing restrictions. Am J Emerg Med.

[12] Dong KA, Lavergne KJ, Salvalaggio G (2021). Emergency physician perspectives on initiating buprenorphine/naloxone in the emergency department: a qualitative study. J Am Coll Emerg Physicians Open.

[13] Kosobuski L, O'Donnell C, Koh-Knox Sharp CP, Chen N, Palombi L (2022). The role of the pharmacist in combating the opioid crisis: an update. Subst Abuse Rehabil.

[14] Harris PA, Taylor R, Thielke R, Payne J, Gonzalez N, Conde JG (2009). Research electronic data capture (REDCap)—a metadata-driven methodology and workflow process for providing translational research informatics support. J Biomed Inform.

[15] Harris PA, Taylor R, Minor BL (2019). The REDCap consortium: building an international community of software platform partners. J Biomed Inform.

